# Dual disorders in liaison-consultation psychiatry. A descriptive study of patients with substance use disorder admitted to a general hospital

**DOI:** 10.1192/j.eurpsy.2022.248

**Published:** 2022-09-01

**Authors:** F. Dinamarca Cáceres, M. Garcia, F. Casanovas, S. Oller Canet, R. Sauras, F. Fonseca, M. Torrens

**Affiliations:** Hospital del Mar, Institut De Neuropsiquiatria I Addiccions (inad), Barcelona, Spain

**Keywords:** Addiction, dual disorder, liaison

## Abstract

**Introduction:**

Several studies describe that the coexistence of a substance use disorder with another psychiatric condition or “dual disorder” (DD) is associated with a worse evolution at all levels, including a greater burden of medical illnesses and greater mortality.

**Objectives:**

To describe the presence of DD and related factors in patients admitted to a General Hospital that required assessment by a psychiatry service.

**Methods:**

A descriptive study that includes patients admitted to the Hospital del Mar in Barcelona for all medical-surgical reasons and attended by the specific addiction psychiatry consultation service between January 2016 and October 2021. Sociodemographic and clinical data are collected including the history of consumption and the diagnosis of dual disorder. Chi-square test was used for comparison between groups.

**Results:**

The sample was 1796 patients (Women: 345. Mean age: 50.3 years; SD: 12.6). 43.7% of the sample presented DD, with axis 1 disorders being the most frequent. There was an association of DD to factors as: being woman (54 vs 41.2% p <0.001), HIV positive serologies (54 vs 42.7% p <0.001), being homeless (49 vs 31.7% p <0.001) and cocaine consumption compared to other substances (53.4 vs 39.8% p <0.001).

**Conclusions:**

In our sample, almost half of patients had DD. The representation of women was significantly lower, however they presented a higher proportion of DD. In this study we describe an association of DD with other biopsychosocial problems, and further studies are necessary to determine in which sense they are related and optimize patient care.

**Disclosure:**

No significant relationships.

